# Do Larval Supply and Recruitment Vary among Chemosynthetic Environments of the Deep Sea?

**DOI:** 10.1371/journal.pone.0011646

**Published:** 2010-07-19

**Authors:** Anna Metaxas, Noreen E. Kelly

**Affiliations:** 1 Department of Oceanography, Dalhousie University, Halifax, Nova Scotia, Canada; 2 Department of Biology, York University, Toronto, Ontario, Canada; National Institute of Water & Atmospheric Research, New Zealand

## Abstract

**Background:**

The biological communities that inhabit chemosynthetic environments exist in an ephemeral and patchily distributed habitat with unique physicochemical properties that lead to high endemicity. Consequently, the maintenance and recovery from perturbation of the populations in these habitats is, arguably, mainly regulated by larval supply and recruitment.

**Methodology/Principal Findings:**

We use data from the published scientific literature to: (1) compare the magnitudes of and variability in larval supply and settlement and recruitment at hydrothermal vents, seeps, and whale, wood and kelp falls; (2) explore factors that affect these life history processes, when information is available; and (3) explore taxonomic affinities in the recruit assemblages of the different chemosynthetic habitats, using multivariate statistical techniques. Larval supply at vents can vary across segments by several orders of magnitude for gastropods; for bivalves, supply is similar at vents on different segments, and at cold seeps. The limited information on larval development suggests that dispersal potential may be highest for molluscs from cold seeps, intermediate for siboglinids at vents and lowest for the whale-bone siboglinid *Osedax*. Settlement is poorly studied and only at vents and seeps, but tends to be highest near an active source of emanating fluid in both habitats. Rate of recruitment at vents is more variable among studies within a segment than among segments. Across different chemosynthetic habitats, recruitment rate of bivalves is much more variable than that of gastropods and polychaetes. Total recruitment rate ranges only between 0.1 and 1 ind dm^−2^ d^−1^ across all chemosynthetic habitats, falling above rates in the non-reducing deep sea. The recruit assemblages at vents, seeps and kelp falls have lower taxonomic breadth, and include more families and genera that have many species more closely related to each other than those at whale and wood falls. Vents also have the most uneven taxonomic structure, with fewer recruits represented by higher taxonomic levels (phyla, orders, classes) compared to seeps and wood and kelp falls, whereas the opposite is true at whale falls.

**Conclusions/Significance:**

Based on our evaluation of the literature, the patterns and regulatory factors of the early history processes in chemosynthetic environments in the deep sea remain poorly understood. More research focused on these early life history stages will allow us to make inferences about the ecological and biogeographic linkages among the reducing habitats in the deep sea.

## Introduction

Chemosynthetic environments in the deep sea have only been discovered recently: hydrothermal vents in 1977 [1], cold seeps in 1984 [2] and organic falls in 1989 [3]. Consequently, our knowledge of the biological assemblages that inhabit these environments and the potential ecological, biogeographic and phylogenetic linkages among those in different locations or habitats is limited. For the better known assemblages, high endemicity has been recorded at vents (∼70%; [4], [5]), whale falls [3] and seeps [6]; however, the assemblages at seeps can share a large component of their macrofaunal assemblages with the surrounding non-reducing deep-sea habitats [7].

The biological communities that inhabit chemosynthetic environments face several challenges that arise from the peculiarities of the habitat. Firstly, endemicity is high and relatively few species are specifically adapted to occupy these habitats. Secondly, these reducing environments are generally ephemeral at decadal scales (except seeps) either because they are geologically unstable (vents) or because the chemosynthetic fuelling resource is non-renewable (large organic falls). Thirdly, these habitats are patchily distributed and can be separated by 10 s–1000 s km by habitat unsuitable for the organisms that are adapted to chemosynthetic conditions. Additionally, most of the organisms that inhabit chemosynthetic environments are either sessile (being attached to a substratum) or show limited mobility in their adult life; they rely on planktonic propagules, mainly larvae, to maintain existing populations and to colonize newly opened areas (e.g. after an eruption at a vent or when a whale lands on the ocean floor).

The successful colonization by larval propagules is essential for the establishment and maintenance of populations in these unique environments. There are several different steps involved in the colonization process: the release of gametes by reproductive adults (fertilization may be internal or external); larval dispersal in the water column and delivery to suitable habitats; larval settlement and metamorphosis; and lastly, survival, growth and recruitment to the adult population. At each step, several different chemical, physical and biological factors drive the selective survival of certain individuals or species, depending on local conditions.

Despite the importance of colonization for populations in ephemeral and unique habitats with high endemism, the process has been poorly studied in chemosynthetic environments. Most research on larval supply, settlement, and recruitment has focused on hydrothermal vents and only in the last decade, and only recently (<5 years) have we begun to examine these processes in cold seeps and organic (whale, wood and kelp) falls.

In this study, we use data from the published scientific literature to compare the different aspects of the colonization process across chemosynthetic habitats. Specifically, we compare the magnitudes of larval supply, settlement, and recruitment at hydrothermal vents, seeps, and whale, wood and kelp falls. We determine the relative amount of variability in the magnitude of these processes at different scales: within studies, among geographic locations, and among chemosynthetic habitats; also, when information is available, we explore factors that affect these processes. For the recruit assemblages only (because of data availability), we explore taxonomic affinities among the different chemosynthetic habitats, using multivariate statistical techniques. We hypothesized that if the assemblages face similar constraints in the different habitats, the rates of larval supply, settlement, and recruitment, as well as the taxonomic diversity of the recruit assemblages, should be similar across habitats. Lastly, we evaluate our ability to draw conclusions on the biogeographical linkages among these habitats based on our current understanding of dispersal potential and colonization specificity, and identify research gaps that need to be addressed.

This study is a synthesis product of the ChEss (Chemosynthetic Ecosystem Science) project of the Census of Marine Life (CoML) program, the main aim of which was to determine the biogeography of deep-water chemosynthetic ecosystems at a global scale and to understand the processes driving these ecosystems. In particular, the stated purpose of CoML was to assess and explain the diversity, distribution, and abundance of marine life and evaluate what is known, unknown, and may never be known about what lives in the global ocean (www.coml.org). Within this context, our study aims to provide an overview of research conducted during the lifetime of CoML (2000–2010) that describes ecological and taxonomic patterns in larval supply, settlement and recruitment in chemosynthetic environments.

## Methods

We obtained all available measures of rates of larval supply, settlement and recruitment, from the published scientific literature, as well as from some recent, unpublished studies, that fit our criteria as described below for each process (Table S1). To minimize variability in sampling approaches, we selected quantitative data from each study that were collected using comparable sampling schemes. For recruit assemblages only, we also explored patterns of biodiversity and taxonomic affinities among the different chemosynthetic habitats, using both the quantitative studies used for the calculation of recruitment rate, as well as several other studies that only contained qualitative data (i.e. not on an area basis) on recruitment (Table S1).

### Larval supply

For rates of larval supply, we included studies that used both larval tubes and sediment traps deployed for 4–15 d (at vents on Juan de Fuca Ridge, East Pacific Rise and Mid Atlantic Ridge), except for the single study done in cold seeps (in the Gulf of Mexico) which lasted 240–270 d. Because all studies at vents (Table S1) included gastropods, this taxon was used for comparisons across segments. However, since only bivalves were collected at seeps (*“Bathymodiolus” childressi*), we used this taxon to compare rates between chemosynthetic habitats (vents vs. seeps). For this comparison, we excluded one study [8], which only reported results on gastropods.

### Settlement

Rates of settlement are difficult to obtain in chemosynthetic environments (or anywhere in the deep sea) because sampling frequency is usually low, and over periods that are too long for the measurement of this process. Only two studies have reported settlement and these only for gastropods (Table S1). Although these two studies were monitoring recruitment rates on deployed substrates over long periods of time (months), the settler stage of gastropods is morphologically distinct, allowing the quantification of settlement on an area basis. Both studies measured settlement at locations with different vent activities (warm and cool vents [9]; at <1 m and ∼10 m from the vent orifice [10]), allowing comparisons between vent habitats and fluid flow regimes.

### Recruitment

#### Patterns in the magnitude of recruitment rate

Recruitment has been measured at different chemosynthetic habitats (vents, seeps, wood kelp and whale falls) and at several locations. We define recruits (termed colonists in some studies) as post-metamorphic individuals that have survived a period of high mortality immediately after settlement until, at least, the sampling time. We do not distinguish recruits based on size or reproductive maturity although, given the length of the sampling intervals, most individuals are presumed to be juveniles. We included in our analyses all studies in which the total number of recruits was counted over a known surface area and period of time, allowing us to calculate the rate of recruitment per unit area per unit time: (i) after an eruption which had eliminated all existing fauna [11], [12]; (ii) following clearance experiments [13]; and (iii) on deployed colonization substrata (all remaining studies in Table S1: “Recruitment”). Although we did not discriminate studies on the basis of the spatial unit used (e.g. colonization panels vs. sediment cores), we only included measures collected over periods of 6–18 months for three reasons. (1) A minimum period of 6 months was used to minimize the possible effects of potentially insufficient conditioning of the deployed substrata on the magnitudes of settlement and recruitment. (The only exception is the inclusion of the single study available from 21°N on the East Pacific Rise, which only lasted for 26 d [14].). (2) A maximum period of 18 months was used to ensure that the observation period was much shorter than the lifetimes of both the habitat and the colonists. (3) This particular temporal range allowed us to include sufficient studies from each type of chemosynthetic habitat to make meaningful comparisons among habitat types. The remote location of these habitats usually does not allow sampling at sub-annual frequencies, constraining us to include measures taken at intervals as long as 18 months.

Average recruitment rates were calculated for vent habitats on the Juan de Fuca Ridge, Galapagos Rift and East Pacific Rise, for each of total macrofauna, gastropods, bivalves and polychaetes. For Galapagos Rift, we calculated a single rate for molluscs rather than separate ones for gastropods and bivalves because of data availability [14]. We explored variability in recruitment rates among studies within segments (Juan de Fuca Ridge and East Pacific Rise) for total macrofauna and each of the 3 classes separately, as well as for the two numerically dominant colonists, the limpet *Lepetodrilus* spp. and the polychaete *Amphisamytha galapagensis*. As for settlement, several studies measured recruitment at locations within a vent field with different fluid flows, allowing us to compare rates between vent habitats.

A number of studies have quantified recruitment rate on wood in six different locations in the world's oceans (Table S1). All used blocks of wood deployed for known periods of time, and all measured recruitment on wood panels, except for [15] who measured recruitment in the sediment underlying the wood fall. The common targeted taxon across most studies, and the one we used for comparisons among sites/studies, was bivalves.

A few additional studies measured recruitment on whale bones or in the sediment immediately beneath a whale fall and a single study measured recruitment in the sediment underlying a kelp fall (Table S1). This enabled us to compare recruitment rates across several chemosynthetic habitats (vents, seeps, wood falls, kelp falls, whale falls) for total macrofauna, gastropods, bivalves and polychaetes.

#### Taxonomic comparisons of recruits among habitats

We created a recruit faunal list for each habitat type (vents, seeps, whale, wood and kelp falls), using data on recruitment collected over a period of 6–18 months. Taxonomic affinities of the recruits among chemosynthetic habitats were explored in two ways. First, we created a contingency table from the recruit faunal lists, examining the shared taxa across more than one chemosynthetic habitat, on the Species, Genus, and Family levels. Second, we conducted a taxonomic distinctness analysis, which is useful for comparing diversity across studies with uncontrolled, unequal, or unknown degrees of sampling effort, and where quantitative data are not available and samples consist of a species list [16], [17]. To examine whether biodiversity structure of the recruit assemblages is similar among chemosynthetic habitats, we used two measures of taxonomic distinctness, which are based on tracing the path through the taxonomic classification tree: (1) average taxonomic distinctness (Δ^+^), the average path length through the taxonomic tree connecting every pair of species in the list, which measures the average degree to which individuals in an assemblage are related to each other [16]; and (2) variation in taxonomic distinctness (Λ^+^), the variance of the taxonomic distance between each pair of species about their mean value Δ^+^, which reflects the unevenness of the taxonomic tree [17]. To create a classification tree for the recruit faunal list from each chemosynthetic habitat, we followed the classification provided by WoRMS (World Register of Marine Species; http://www.marinespecies.org). When a taxon was not listed on WoRMS, we further consulted the taxonomic information provided by ChEssBase (http://www.noc.soton.ac.uk/chess/database/db_home.php). Using these data we generated a master list that included the chemosynthetic recruit fauna from all habitat types combined. For each chemosynthetic habitat, we examined whether the taxonomic distinctness measures (Δ^+^, Λ^+^) of the recruit faunal list fell within the confidence limits generated by 1000 simulations of random subsets of *m* species from the master list [16]. These randomization procedures test the null hypothesis that a faunal list from one habitat type has the same taxonomic structure (i.e. diversity) as the master list. We conducted similar analyses to compare the vent recruit fauna among Eastern Pacific Vent Segments (e.g. Juan de Fuca Ridge, East Pacific Rise, Galapagos Rift) using: (1) a master list comprised of all recruit fauna from this region; and (2) a master list comprised of all the chemosynthetic fauna found at these locations (i.e. our recruit fauna plus all adult fauna reported at these locations), which was generated using ChEssBase. All taxonomic distinctness analyses were conducted using PRIMER (Version 6, PRIMER-E Ltd).

## Results

### Larval supply

**Figure 1:**
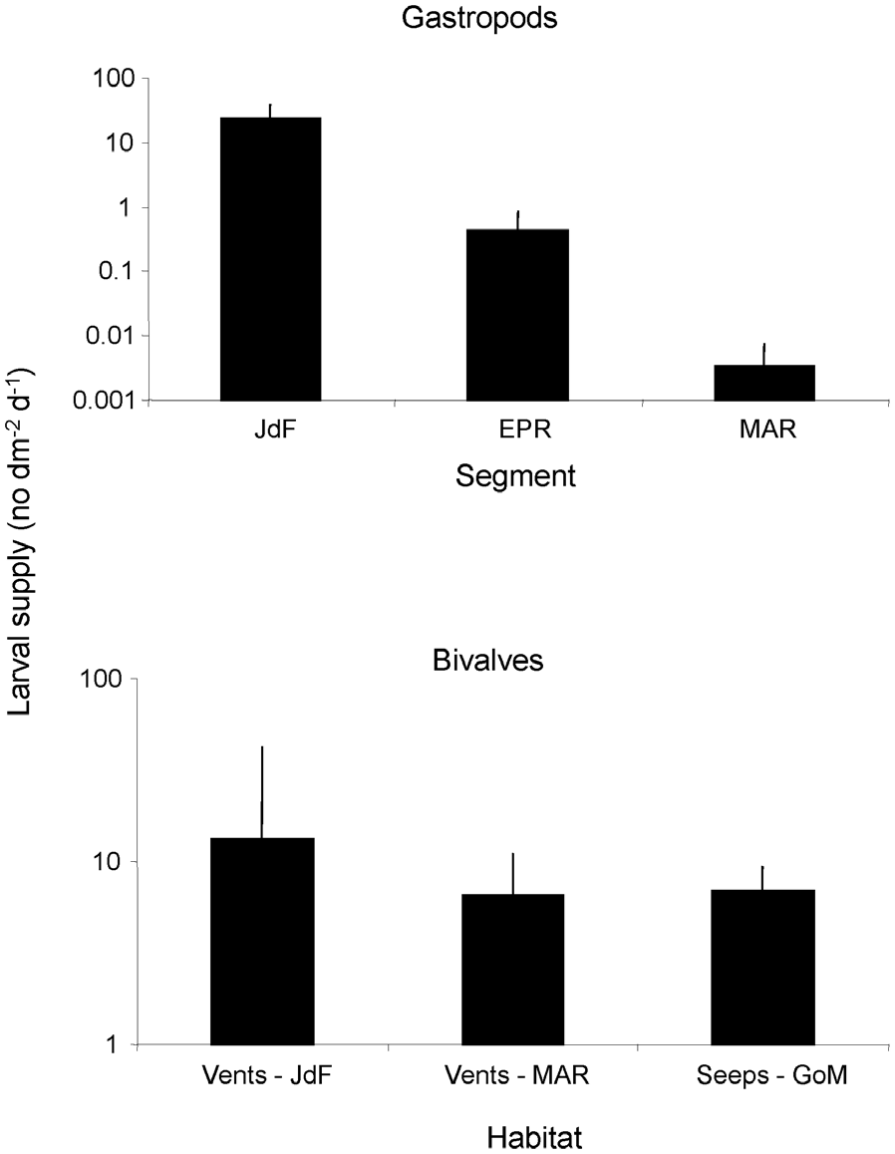
Larval supply of gastropods (+SD) and bivalves in chemosynthetic habitats. Rates for gastropods were measured at vent habitats on the Juan de Fuca Ridge (JdF, [18]), East Pacific Rise (EPR, [8]) and Mid Atlantic Ridge (MAR, [42]); and rates of bivalves at vents (JdF, MAR) and seeps in the Gulf of Mexico (GoM, [25]). Source of variation is within each study.

Although poorly studied in chemosynthetic environments, the supply of individuals to the benthos where they can settle and metamorphose is linked to larval abundance in the water column [18]. In the 4 studies that have measured larval supply at hydrothermal vents, gastropods and bivalves are the most abundant taxa. Larval supply of gastropods (the most abundant being *Lepetodrilus fucensis* and *Depressigyra globulus* on the Juan de Fuca Ridge, and *Cyathermia naticoides* and *Lepetodrilus* spp. on the East Pacific Rise) can vary by several orders of magnitude across segments (Fig. 1). In contrast, larval supply of bivalves is similar (∼10 individuals dm^−2^ d^−1^) both at vents on different segments and at cold seeps (Fig. 1).

### Settlement

**Figure 2:**
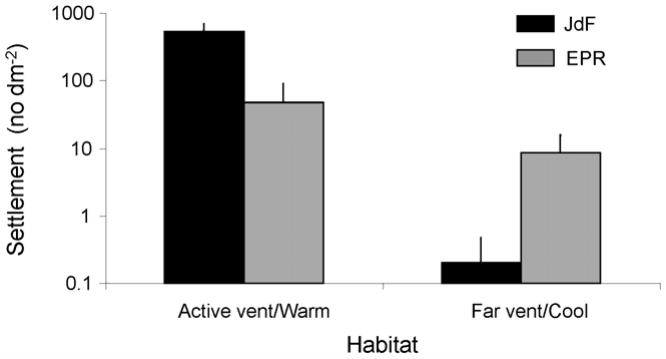
Patterns in settlement of gastropods (+SD) at vents. These measures were taken using colonization plates placed within 0.5 m of the orifice of an active vent (Juan de Fuca Ridge, [10]) or at warm vents (East Pacific Rise, [9]) and at ∼10 m from an active vent (Juan de Fuca Ridge, [10]) or at cool vents (East Pacific Rise, [9]). Source of variation is within each study.

Quantitative measures of settlement have only been obtained for gastropods at hydrothermal vents in two studies (East Pacific Rise [9]; Juan de Fuca Ridge:[10]), because this taxon has a morphologically distinct settler stage (a protoconch). Both these studies measured settlement in habitats that varied in vigour of flow of the hydrothermal fluid (warm or on-vent vs. cool or 10 s of meters from a vent opening). Although settlement varied by 2 orders of magnitude between the two studies, it was greater under more vigorous fluid flow for both studies (Fig. 2).

### Recruitment

#### Patterns in the magnitude of recruitment rate

**Figure 3:**
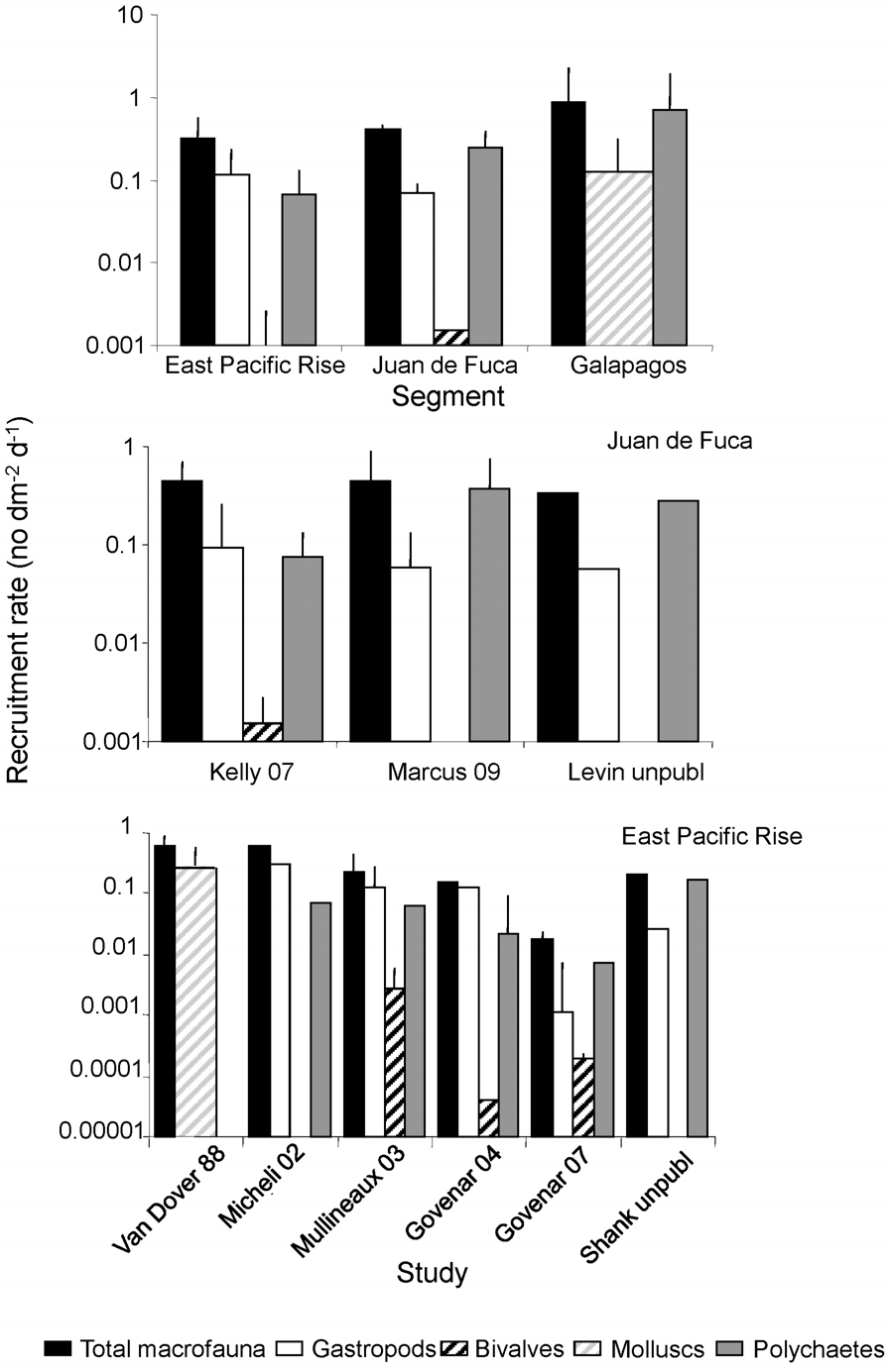
Rate of recruitment (+SD) of three invertebrate families and of total macrofauna at vents. Rates are estimated based on the deployment of substrates for known periods of time at the Juan de Fuca Ridge and East Pacific Rise. Top panel shows rates averaged across all studies for each segment. Middle and bottom panels show rates averaged for each study. Van Dover et al. (1988) only provided combined rates for molluscs (instead of separate rates for gastropods and bivalves; wider bar denotes this) for both Galapagos Rift (top panel) and East Pacific Rise (bottom panel).

**Figure 4:**
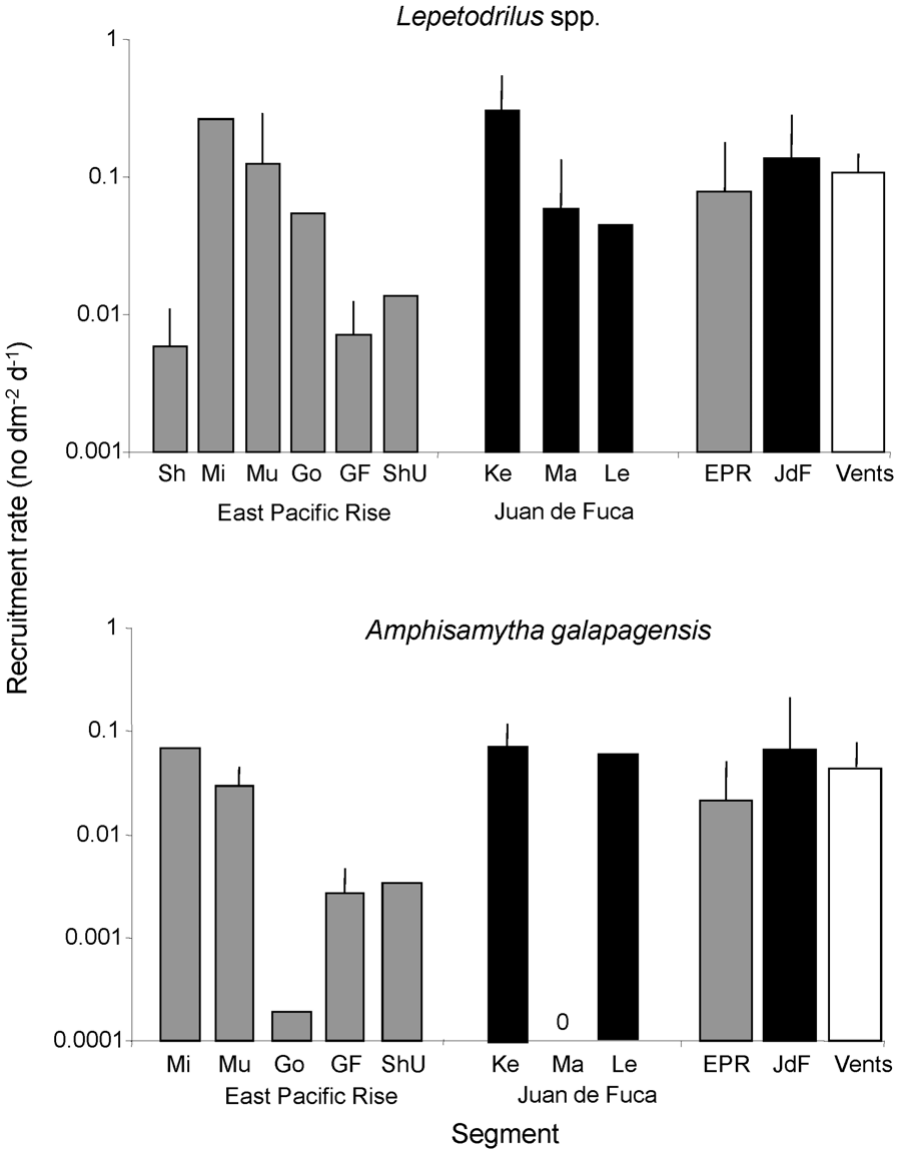
Rate of recruitment (+SD) of two dominant taxa at vents. The taxa are the gastropod genus *Lepetodrilus* and the polychaete *Amphisamytha galapagensis*, shown for different studies at the Juan de Fuca Ridge (JDF)and East Pacific Rise (EPR), as well as averaged across studies for each segment and across both segments for the vent habitat. EPR: Sh = [11]; Mi = [31]; Mu = [30]; Go = [13]; GF = [19]; ShU = Shank unpublished data; JDF: Ke = [10]; Ma = [12]; Le = Levin unpublished data.

Several studies have measured recruitment rates at hydrothermal vents on the Juan de Fuca Ridge, East Pacific Rise and Galapagos Rift, using colonization plates or blocks deployed for known periods of time (Fig. 3). On the Juan de Fuca Ridge, recruitment rate is highest for gastropods or polychaetes (depending on the study), although high (order of magnitude) variability (spatial and temporal) has been observed within studies and, for polychaetes, across studies (Fig. 3). At the East Pacific Rise, rates have been as variable as at Juan de Fuca Ridge, both within and across studies for all taxa. Recruitment rate of the two numerically dominant species can vary by several orders of magnitude among studies within a segment, but is less variable among segments, when averaged across studies (Fig. 4). Interestingly, total recruitment rates (calculated for all individuals of all taxa in an assemblage of different taxonomic groups) are remarkably consistent, and range between 0.1 and 1 individual dm^−2^ d^−1^, across all studies (except [19]) and when averaged across segments (Fig. 3).

**Figure 5:**
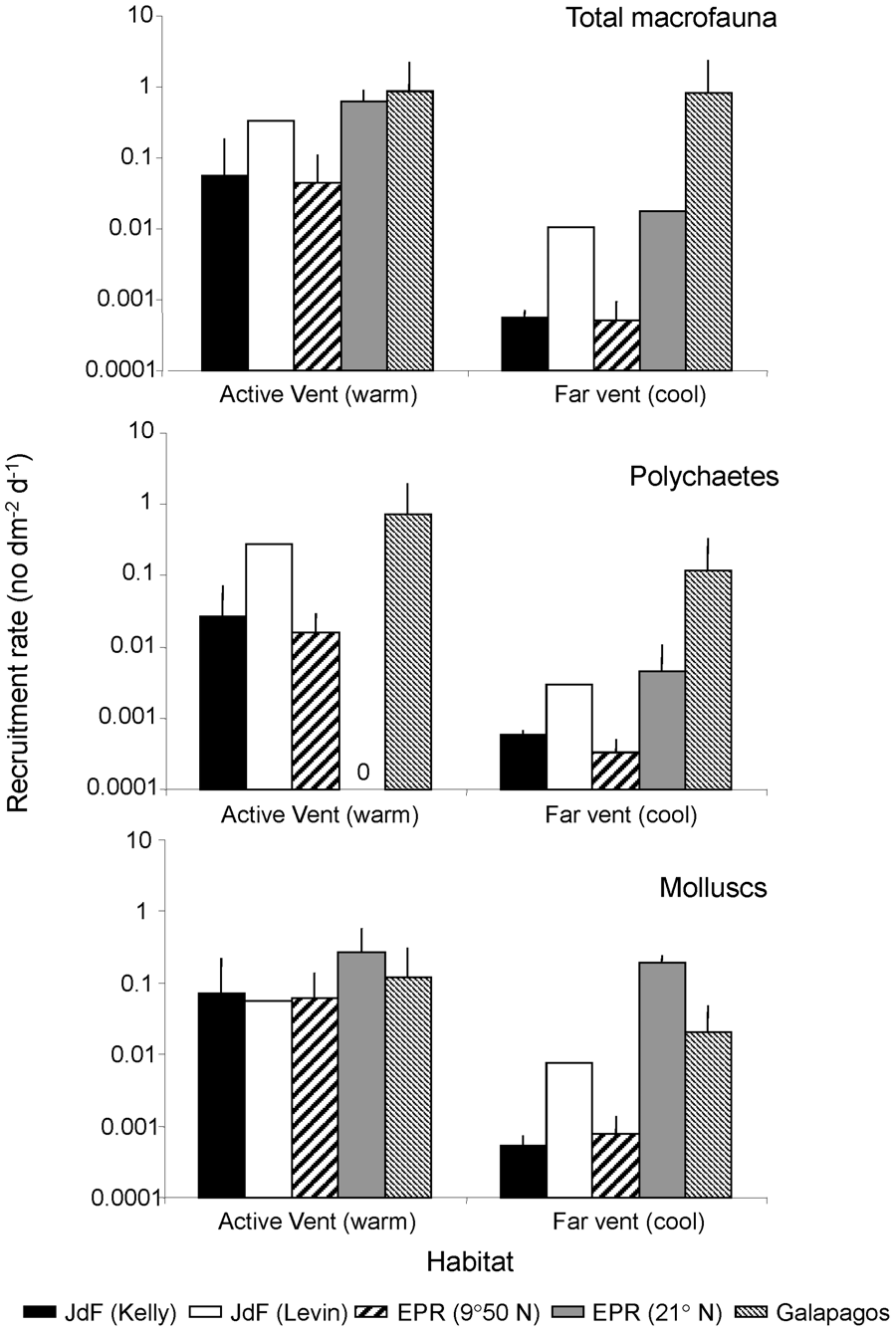
Rate of recruitment of total macrofauna, polychaetes and molluscs (+SD) at different vent habitats. These rates are measured near the orifice of an active vent or at vents with warm fluids, and at ∼10 m from an active vent or at vents with cool fluids. Data are from: JdF(Kelly) = [10]; JdF(Levin) = Levin unpublished data; EPR(9°50) = [30]; EPR(21°), Galapagos = [14].

As for settlement, recruitment rate within a vent field varies with flow vigour. For total macrofauna, polychaetes and molluscs, recruitment rate is greater near (<1 m) than far from a vent opening, and is also greater in vents with warmer fluid (Fig. 5). This is most likely the outcome of post-settlement processes that affect survival and perhaps movement, rather than a response to a cue.

**Figure 6:**
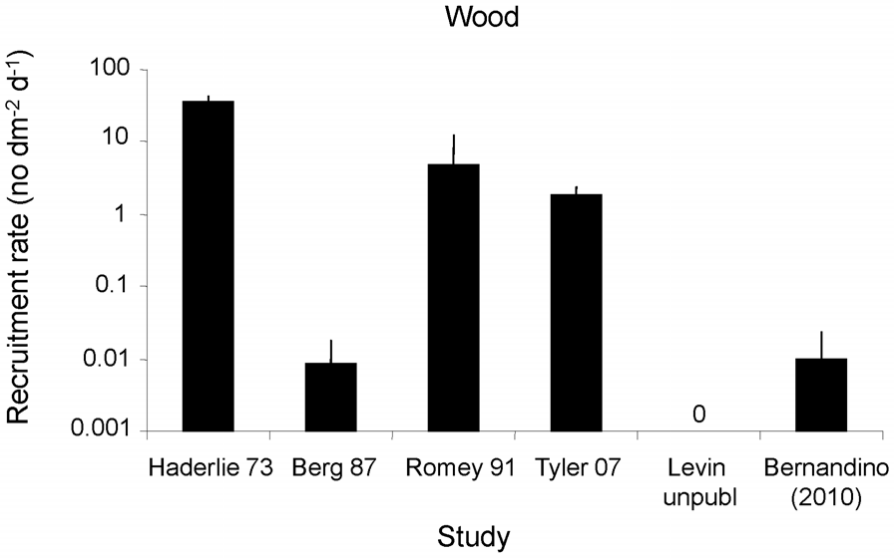
Rate of recruitment of bivalves (+SD) on wood in different studies. [43]: *Xylophaga washingtonia*; [44], [45]: *X. atlantica*; [46]: *X. depalmai*; [15]: Bivalve unid. Q and Thysaridae sp. 1.

A few studies have examined recruitment of different taxa on wood, and bivalves have been the most frequently recorded taxon. These bivalves include mostly wood specialists, such as species belonging to the genus *Xylophaga*. Recruitment rates of bivalves have been quite variable (ranging from 0.009 to 38 individuals dm^−2^ d^−1^), most likely reflecting variability in the available larval pool, the period of observation, and characteristics of the substratum (Fig. 6). However, these rates are much higher than at vents, implying high larval availability of these specialists in the water column at all times.

**Figure 7:**
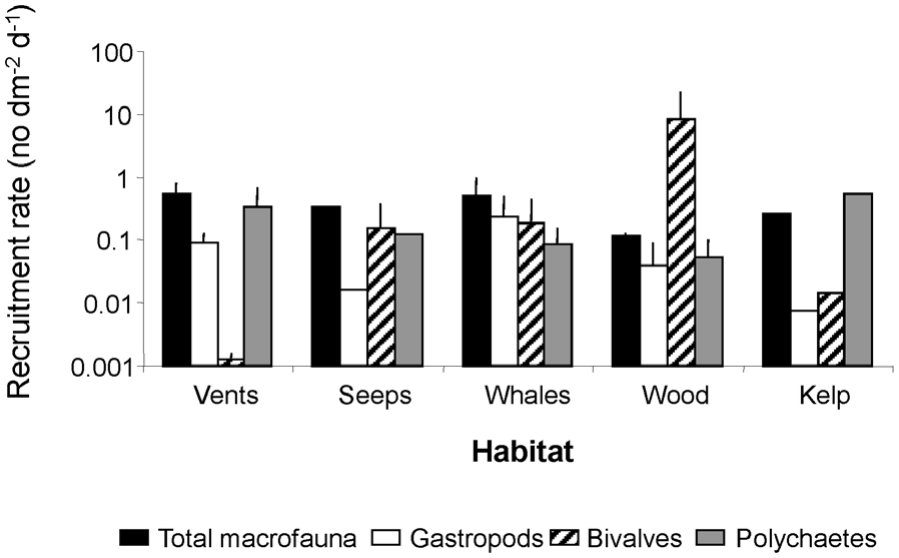
Rate of recruitment (+SD) of three invertebrate families and of total macrofauna at five different chemosynthetic habitats. The source of variation is among studies at each habitat.

When compared across different chemosynthetic habitats, recruitment rates of gastropods and polychaetes vary by 1–2 orders of magnitude, whereas that of bivalves varies by 5 orders of magnitude (Fig. 7). This is not surprising given the high specialization of the wood boring bivalves to rapidly and effectively colonize an ephemeral resource. Interestingly, total recruitment rate again only ranges between 0.1 and 1 ind dm^−2^ d^−1^ across all habitats.

#### Taxonomic comparisons of recruits among habitats

The Families, Genera and/or Species of recorded recruits found in more than one chemosynthetic habitat represented four different Phyla (Table 1). While most lower-level taxa (27 taxa) were restricted to only recruiting to two different habitat types, some (12 taxa) occurred in 3 different habitat types, and fewer still (6 taxa) were found to recruit to 4 different habitats. While there were no species common to all 5 habitat types, one Genus, *Ophryotrocha*, has been reported to occur at vents, seeps, and whale, wood and kelp falls. Three polychaete families were common in all 5 habitat types: Spionidae, Dorvilleidae, and Ampharetidae. For the higher-level taxa, only the phyla Annelida (Class Polychaeta: Orders Eunicida, Phyllodocida, Spionida, Terebellida) and Mollusca (Class Gastropoda) had faunal representatives common to all habitats. By far, polychaetes were the most widespread taxa across all habitat types, whereas the occurrence of arthropods and molluscs was reported most frequently at vents, and whale and wood falls (Table 1).

**Table 1:** Taxonomic list of shared Species, Genera, or Families of recruits within 4 phyla present in more than one chemosynthetic habitat.

Taxonomy			Habitat				
Phylum	Family	Genus or Species	Kelp	Seep	Vent	Whale	Wood
Annelida	Alvinellidae	*Paralvinella palmiformis*			X		X
	Ampharetidae	*Amphisamytha galapagensis*		X	X	X	X
		*Samytha californiensis*	X				X
	Amphinomidae			X		X	
	Capitellidae			X	X	X	X
	Cirratulidae			X		X	X
		*Chaetozone*	X	X			X
		*Monticellina*	X				X
	Dorvilleidae	*Ophryotrocha*	X	X	X	X	X
		*Parougia*		X	X		X
	Glyceridae			X	X	X	
	Hesionidae		X		X		X
	Lumbrineridae			X		X	
	Maldanidae	*Nicomache*		X	X	X	X
	Nephtyidae			X		X	
	Nereididae			X	X	X	
		*Nereis*		X	X		
	Opheliidae			X		X	
	Orbiniidae				X	X	X
	Phyllodocidae			X	X	X	
	Polynoidae		X		X	X	X
		*Harmothoe*	X		X		
	Serpulidae				X		X
	Siboglinidae	*Ridgeia piscesae*			X	X	X
	Spionidae		X	X	X	X	X
		*Prionospio*		X	X		
	Syllidae			X	X	X	X
		*Sphaerosyllis*		X	X		
	Terebellidae			X	X		
Arthropoda	Ammotheidae	*Ammothea verenae*			X		X
		*Sericosura*			X	X	
	Caprellidae					X	X
	Gammaridae					X	X
	Nannastacidae	*Cumella*				X	X
Cnidaria	Metridiidae	*Metridium*				X	X
Mollusca	Buccinidae	*Buccinum*			X	X	
	Hyalogyrinidae	*Hyalogyrina*	X		X	X	
	Lepetodrilidae	*Lepetodrilus fucensis*			X	X	X
	Mytilidae	*Adipicola*				X	X
		*Bathymodiolus*		X	X		
		*Idasola*			X		X
	Peltospiridae	*Depressigyra globulus*			X	X	X
	Provannidae	*Provanna variabilis*		X	X	X	X
	Simrothiellidae	*Helicoradomenia*			X		X
	Vesicomyidae	*Calyptogena*		X	X		
	Thyasiridae					X	X
	Turridae	*Phymorhynchus major*		X	X		

**Figure 8:**
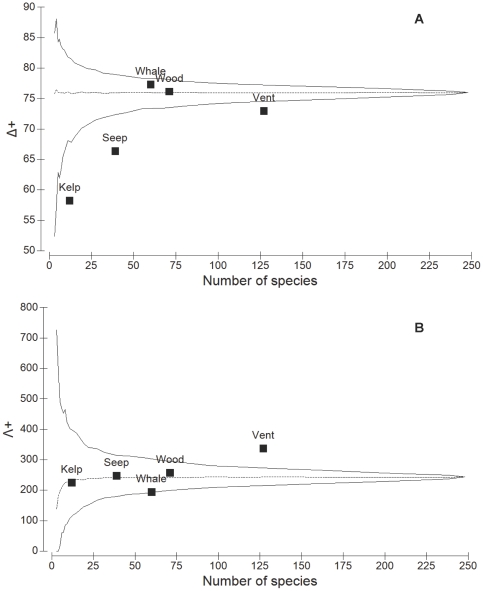
Taxonomic comparisons of the recruit assemblages among chemosynthetic habitats. (A) Average taxonomic distinctness (Δ^+^) and (B) variation in taxonomic distinctness (Λ^+^) for the recruit faunal lists from the five different chemosynthetic habitat types (Kelp, Seep, Vent, Whale, Wood) by numbers of species in each list. Dotted central line is (A) average taxonomic distinctness and (B) variation in taxonomic distinctness for the master list, generated from the full list of all chemosynthetic recruit fauna from all habitat types combined. Funnel (solid) lines are 95% simulated confidence limits for random selections of subsets of *m* species from the master list.

The recruit fauna at vents, seeps and kelp falls had significantly lower than expected (if they were identical to the master list) average taxonomic distinctness (Δ^+^) (i.e. lower taxonomic breadth or diversity) compared to the recruits from whale and wood falls (Fig. 8a). This result suggests that the recruit assemblages at vents, seeps and kelp falls include more lower-level taxa (i.e. families, genera) that are more species rich (i.e. they include many species more closely related to each other) than those at whale and wood falls. In addition, variation in distinctness (Λ^+^) was higher than expected at vents (i.e. representing a more uneven taxonomic tree) (Fig. 8b), suggesting that the higher taxonomic levels (e.g. phyla, orders, classes) present contain fewer species in these habitats than in seeps and wood and kelp falls. The opposite was observed for the assemblages at whale falls.

**Figure 9:**
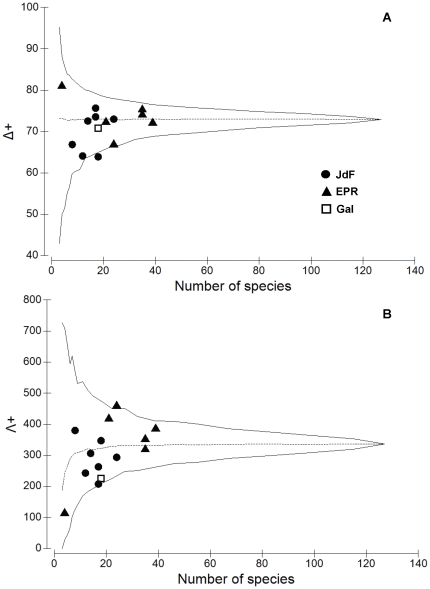
Taxonomic comparisons of the recruit assemblages among vents in the Eastern Pacific. (A) Average taxonomic distinctness (Δ^+^) and (B) variation in taxonomic distinctness (Λ^+^) for the 14 recruit faunal lists from Vent habitats of the Eastern Pacific (JdF, Juan de Fuca Ridge; EPR, East Pacific Rise; Gal, Galapagos Rift) plotted against the number of species in each list. Dotted horizontal line is (A) average taxonomic distinctness and (B) variation in taxonomic distinctness for the master list, generated from the full list of all recruit fauna combined from all Vent Habitats in the Eastern Pacific. Funnel lines are 95% simulated confidence limits.

**Figure 10:**
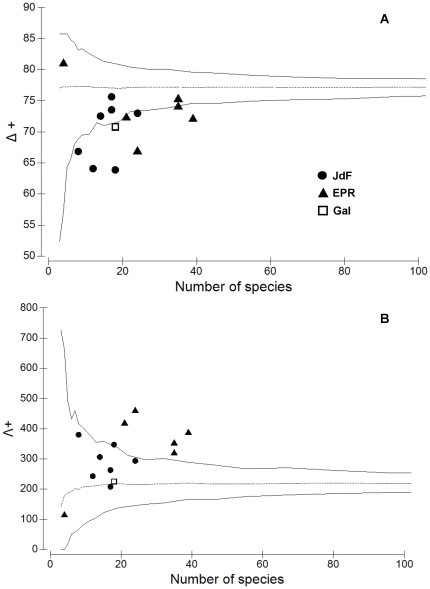
Taxonomic comparisons between the recruit and mature assemblages at vents in the Eastern Pacific. (A) Average taxonomic distinctness (Δ^+^) and (B) variation in taxonomic distinctness (Λ^+^) for the 14 recruit faunal lists from Vent habitats of the Eastern Pacific (values and abbreviations as in Fig. 9). Dotted horizontal line is (A) average taxonomic distinctness and (B) variation in taxonomic distinctness for the master list, generated from the full list of all mature fauna (>2–3 years old) combined from all Vent Habitats for these same sites in the Eastern Pacific. Funnel lines are 95% simulated confidence limits.

For hydrothermal vents on the Eastern Pacific, average taxonomic distinctness (Δ^+^) and variation in taxonomic distinctness (Λ^+^) of the recruit fauna were not significantly different from expectation, except for one study from Juan de Fuca Ridge and another from East Pacific Rise (Fig. 9). These results suggest that most recruit faunal assemblages in the Eastern Pacific have a similar taxonomic structure (i.e. diversity). In contrast, when compared to a master list generated from the entire mature assemblage at vents on the Eastern Pacific (i.e. all fauna from Vent assemblages >2–3 years old; the dotted line in Fig. 10a), most recruit faunal assemblages (6–18 mo old) are less diverse (Fig. 10a). Specifically, at the East Pacific Rise, most recruit faunal assemblages have higher than expected variation in taxonomic distinctness (Λ^+^) (Fig. 10b), suggesting very uneven taxonomic trees compared to a mature vent assemblage.

## Discussion

### Larval supply

Larval supply to the benthos has been measured at both vents and seeps, but only in a few studies. At vents, it is directly linked to the hydrodynamic regime, which can result in homogeneous supply within a vent field [18] or give rise to site-specific patterns [8]. The difference in the level of variability in supply between gastropods (high variability across segments) and bivalves (similar magnitude in vents and seeps) may be related to the planktonic larval duration. A longer planktonic duration, as evidenced for, at least, seep bivalves, would tend to reduce variability that may arise from shorter term fluctuations in the hydrodynamic regime, timing of spawning, or in mortality rate.

Dispersal potential has been assessed for a handful of species that inhabit vents, based on experiments on larval development and behaviour, and local currents. For example, embryos of the tubeworm *Riftia pachyptila* become ciliated larvae over a developmental period of ∼20 d [20]. These larvae can potentially survive in the water column for 38 d, based on the energy reserves of the egg and the energy requirements for respiration [21]. Over a period of 20 d, it is possible for them to disperse up to 90 km along the axial valley [21], which would allow them to reach vent fields other than their origin. Interestingly, the embryos of both *Riftia pachyptila* and the polychaete *Alvinella pompejana* show successful development in temperatures that are generally cooler than in the parental habitat [20], [22]. Both these studies suggested that their results were indicative of dispersal away from the vent habitat during the larval period, at least for these two species. A similar conclusion was reached in [23], who collected postlarval shrimp from a hydrothermal vent in the water column several 100 s of meters above the bottom.

Developmental studies done with invertebrates that inhabit cold seeps are rare, but are suggestive of a greater dispersal potential for seep than vent species. For example, larvae of the gastropod *Bathynerita naticoidea* and the bivalve *“Bathymodiolus” childressi* have been collected in the top 100 m of the water column above the Brine Pool in the Gulf of Mexico [24], [25]. Veligers of *“Bathymodiolus” childressi* develop within 8 d of fertilization, and planktonic larval duration has been estimated from 3 to 13 months [26]. Hatched larvae of *B. naticoidea* survived in the laboratory for at least 90 d and may have a planktonic duration of up to 12 months [24]. These long planktonic periods would allow for great dispersal distances, possibly outside the Gulf of Mexico under favourable currents.

For organic falls, a single study has examined larval development in the bone-eating worm of the genus *Osedax* [27]. In contrast to species from vents and seeps, development of *Osedax* is fast and the trochophore larvae can settle as early as 10 d after spawning, although settlement was observed as late as 16 d after spawning. These results imply shorter dispersal distances for this species than for species from vents and seeps; however, it has been suggested that nearest neighbour distances of whale falls can also be quite short [3] and the short development time and consequent planktonic period may not restrict colonization of newly-opened habitats.

The patterns of larval supply remain largely unknown in reducing environments. The magnitude of larval availability will determine whether a newly opened habitat (e.g. new vent, organic fall) will be colonized and at what rate, and whether existing populations act as sources or sinks of individuals. Larval collection and identification can allow us to determine the extent to which different reducing habitats can act as stepping-stones of dispersal on ecological time scales. In combination, these measures will enable us to obtain estimates of population connectivity across different chemosynthetic habitats, as well as generate hypotheses about biogeographic linkages. The quantification of larval supply to the benthos is not logistically difficult, particularly near the larval origin, and traditional methods such as net tows and sediment traps have been used successfully (but see [28]). It is therefore a logical and tractable direction in which research in reducing environments can proceed in the next decade.

### Settlement

The logistical difficulties of measuring settlement in deep-sea habitats (including reducing ones) have precluded general conclusions on the patterns and mechanisms of this process. The opportunistic results that have been obtained to date indicate that settlement is greatly influenced by the chemical characteristics of the environment. Studies at vents have shown that settlement is greater near active venting, indicated by vigorous flow and/or high temperature of the emanating fluid, than away from flow. Similarly for cold seeps, settlers of *“Bathymodiolus” childressi* tended to be more abundant in the inner than the outer zone of the Brine Pool, Gulf of Mexico [25], where presumably the influence of methane seepage was greatest. These spatial patterns in settlement at both vents and seeps likely result from either differential settlement in response to certain chemical cues present in the emanating fluid or differential survival during settlement because of variability in habitat suitability (e.g. resource availability, such as in the physical and chemical substrates or of symbionts). Presently, the relative importance of the chemical characteristics of the vent fluid and of the microbial fauna present on the available substratum is being explored in experiments conducted at the East Pacific Rise (T Shank, Woods Hole Oceanographic Institution, personal communication).

### Recruitment

Recruitment is the best studied process of colonization to date, and has been measured in different chemosynthetic habitats at different locations in the world's oceans. Across chemosynthetic habitats, rates of recruitment are relatively similar (within 1–2 orders of magnitude) for most taxa except bivalves. These rates are affected by pre-settlement processes, such as larval supply and settlement, as well as post-settlement mortality. At the recruit stage, predation as well as competition for resources can have pronounced consequences for spatial patterns. The mechanisms that affect recruitment rate in chemosynthetic habitats are poorly known. At hydrothermal vents (where they have been measured), larval supply and settlement are much more variable than recruitment rates, suggesting that post-settlement processes may be responsible for the modulation of recruitment.

The physicochemical characteristics of the environment (and the consequent availability of resources) appear to be primarily responsible for the patterns in recruitment [10], [29], [30], and biological interactions, such as competition and predation, play a secondary role [9], [31], [32]. Given the particular conditions in these environments, it is most likely that recruitment is regulated similarly across chemosynthetic habitats. At vents, rate of recruitment was greater in locations with more vigorous fluid flow for all taxa examined (including total macrofauna). In manipulative experiments using colonization trays with and without added sulfide and placed inside and outside methane seeps, total recruitment rate did not vary among treatments [33]. However, different taxa showed avoidance or attraction to sulfide, while others exhibited greater recruitment rates inside than outside the seeps.

Overall, recruitment rate for total macrofauna consistently ranged between 0.1 and 1 ind dm^−2^ d^−1^ for all chemosynthetic environments. These rates are generally higher than in non-reducing habitats in the deep-sea (which are in the order of 0.001–0.01 ind dm^−2^ d^−1^; e.g. [34], [35], [36]). Larval availability and settlement are likely to differ more widely than <1 order of magnitude among chemosynthetic environments because of differences in habitat distribution, density of local adult populations, hydrodynamics and topographic relief; thus, it is unlikely that these two processes are responsible for the observed low variability in recruitment rate. We propose that the rate of recruitment is similar across reducing habitats and falls within the observed range because it is mainly regulated by resource availability. In non-chemosynthetic environments in the deep sea, the assemblages are mainly food-limited to detrital inputs from allochthonous primary production in shallow water. In contrast, in reducing environments, local carbon production is high, allowing for greater rates of recruitment than in other deep-sea habitats. However, the spatial extent of the reducing chemosynthetic conditions required for growth is limited compared to the vastness of other deep-sea habitats. Although carbon availability per unit area has not been quantified in chemosynthetic habitats to date, it most likely is intermediate to that in other deep-sea habitats and those in shallow water where photosynthesis dominates.

Differences in the taxonomic structure of their recruits existed among all five chemosynthetic habitat types examined. While vents had the highest recruit species richness, they also had low taxonomic diversity. The seep and kelp fall recruits also had lower taxonomic diversity, but higher taxonomic evenness than vents. The fauna recruiting to whale and wood falls appears to have the greatest taxonomic diversity, but whale falls also have more taxa evenly represented across many higher and lower taxonomic levels. It has been suggested that the mature assemblages inhabiting whale bones are the most diverse of any hard-substratum habitat in the deep sea [3]. The greater taxonomic diversity observed at whale falls than at other chemosynthetic habitats relates to a combination of habitat persistence and the provision of a complex suite of niches which promotes the recruitment and coexistence of sulphophiles, opportunistic and other deep-sea species, and whale fall specialists on a single carcass [37]. The chemical conditions at vents and seeps may restrict recruitment to only those organisms physiologically capable of tolerating high concentrations of sulphides, metals, and low oxygen, resulting in low diversity but high endemism [4], [38]. Differences in the rate and mechanisms of sulphide delivery between vent, seep, and whale fall habitats [39] may also be partly responsible for the observed differences in diversity. In addition, the transient nature, small habitat area and restricted distribution of wood and kelp falls in the global ocean likely promotes the recruitment and dominance of highly specialized, fast growing, opportunistic fauna. Overall, the taxonomic structure of recruits at whale falls appears to differ from vents and seeps for reasons other than at wood and kelp falls.

The recruit assemblages of vents on the Eastern Pacific (Juan de Fuca Ridge, East Pacific Rise, Galapagos Rift) share similar levels of taxonomic diversity, but are less diverse and, particularly at the East Pacific Rise, have a highly variable taxonomic structure when compared to mature faunal assemblages at these same sites. This pattern suggests that only a subset of the total fauna at any one vent site colonizes within the timeframe we used (6–18 mo), and is consistent with the current understanding of vent successional dynamics, that mature vent assemblages can take upwards of 2–3 years to amass their entire faunal structure [12], [30], [40]. A detailed comparison of the species richness between recruit and mature faunal assemblages at Axial and Endeavour (Juan de Fuca Ridge), and 9°50′N (East Pacific Rise) vent segments also confirms this pattern: after 12–13 mo, approximately only 35% (61 species), 25% (60 species) and 15% (197 species) of the total faunal assemblage had recruited to these three areas, respectively (percentages based on total species reported in ChEssBase). The greater variation in taxonomic breadth for recruits at the East Pacific Rise also suggests that species do not recruit at the same rate or with the same pattern at vent sites on the East Pacific Rise and Juan de Fuca Ridge.

An important lingering question in the biogeography of chemosynthetic systems is the extent to which species are capable of dispersing among different chemosynthetic habitats [6]. Successful settlement and recruitment of individual dispersing propagules is obviously an important step in determining similarities and/or differences in diversity among habitat types and biogeographic regions. The degree to which chemosynthetic fauna may be able to use different reducing habitats (as dispersal stepping stones; e.g. [41]) also depends on their proximity, availability of ocean currents for transport between habitats, and the nature of the supply of reducing compounds. The roles of larval supply, settlement, and recruitment in maintaining linkages among different chemosynthetic habitats have not been sufficiently investigated to arrive at any definite conclusions to date. In our study, shared taxa of recruits among habitat types occurred mostly at the family level or higher, with little overlap at the genus or species levels, implying a low frequency of dispersal. While vent, whale, and wood fall habitats shared species and genera of recruits more frequently, this similarity amongst habitats was mostly driven by similarities in diversity from colonization experiments conducted on wood and whale bones in close proximity to vent habitats in the Northeast Pacific (Levin unpublished). From these experiments, 5 species were found to recruit to all three habitat types (the gastropods *Lepetodrilus fucensis*, *Depressigyra globulus* and *Provanna variabilis*, the polychaete *Amphisamytha galapagensis*, and the tubeworm *Ridgeia piscesae*), implying that some species can successfully colonize different chemosynthetic habitats as long as dispersal distances are short. However, we must caution that the exclusion of some species/taxa from our study that have only been reported from mature assemblages (i.e. >6–18 mo. old) may underestimate the similarities in taxonomic composition we calculated among the different chemosynthetic habitat types. Based on studies of total fauna (not just recruits), in 2003, only 11 species were known to be shared between sedimented vent sites and whale falls, and 20 species shared between whale falls and seeps [3]. Whale bones share very few species with the surrounding non-reducing deep-sea [3], whereas seep infauna can be very similar to non-seep fauna and include few endemics, particularly in shallow depths [7]. Long-distance dispersal among different chemosynthetic habitat types may be possible, given that the overlap for some fauna at higher taxonomic levels (e.g. at the family level for polychaetes) suggests close evolutionary linkages among habitat types [4]. Future sampling and experimentation at multiple chemosynthetic habitat types will, without a doubt, shed more light onto the roles of larval supply, settlement and recruitment in controlling the patterns of diversity on a global scale.

### Conclusions

In summary, the different stages of the colonization process of benthic marine invertebrates have not been examined to the same extent within or between chemosynthetic habitats, and many gaps remain to be addressed. Larval supply to the benthos has only been measured in hydrothermal vents in 6, and at methane seeps in 2, studies. Based on this scant field data, in combination with a few laboratory studies on larval development, we propose that larval dispersal potential may be greatest for seep species, lower for vent species and lowest for the boneworm. However, field studies that can address this gap are not logistically complicated and can be pursued in a range of habitats and locations. In contrast, settlement is much more logistically difficult to measure and most likely will continue to elude us until high frequency sampling becomes feasible, perhaps through cabled observatories. Recruitment is the best studied stage, both geographically and in different chemosynthetic environments. During the first 1.5 years after the opening of a new habitat, the rate of addition of total new recruits is similar among chemosynthetic environments and higher than that recorded in a few studies in the non-reducing deep sea. The recruit assemblage is taxonomically richest in whale falls, and richer at methane seeps than vents, with very little species overlap among different habitats. It should be noted that the number of studies that have quantitatively measured recruitment rate, although greater than for the other processes, is still quite low, making conclusions premature. Experimental approaches, such as the deployment of colonization substrates over known periods of time and at different spatial designs, can allow us to obtain a better understanding of the patterns and factors that regulate recruitment and thus the persistence of populations in these unique habitats.

## Supporting Information

Table S1 Studies. JdF: Juan de Fuca Ridge; Exp: Explorer Ridge; EPR: East Pacific Rise; GAL: Galapagos Rift; MAR: Mid Atlantic Ridge; Gulf of Mexico; SCB: Santa Cruz Basin; CM: Cape Nomamisaki; MC: Monterey Canyon; NWA: Northwest Atlantic; NEP: Northeast Pacific; mab: metres above bottom; † these additional studies were used for taxonomic comparisons only.
(0.11 MB DOC)

## References

[1] Corliss JB, Dymond J, Gordon LI, Edmond JM, von Herzen RP (1979). Submarine thermal springs on the Galápagos Rift.. Science.

[2] Paull CK, Hecker B, Commeau R, Freeman-Lynde RP, Neumann C (1984). Biological communities at the Florida Escarpment resemble hydrothermal vent taxa.. Science.

[3] Smith CR, Baco A (2003). Ecology of whale falls at the deep-sea floor.. Oceanogr Mar Biol Ann Rev.

[4] Tunnicliffe V, McArthur AG, McHugh D (1998). A biogeographical perspective of the deep-sea hydrothermal vent fauna.. Adv Mar Biol.

[5] Desbruyères D, Segonzac M, Bright M (2006). Handbook of deep-sea hydrothermal vent fauna. Second completely revised edition.. Linz: Denisia.

[6] Cordes EE, Carney SL, Hourdez S, Carney RS, Brooks JM (2007). Cold seeps of the deep Gulf of Mexico: Community structure and biogeographic comparisons to Atlantic equatorial belt seep communities.. Deep-Sea Res I.

[7] Levin LA (2005). Ecology of cold seep sediments: interactions of fauna with flow, chemistry and microbes.. Oceanogr Mar Biol Ann Rev.

[8] Adams DK, Mullineaux LS (2008). Supply of gastropod larvae to hydrothermal vents reflect transport from local larval sources.. Limnol Oceanogr.

[9] Mullineaux LS, Mills SW, Goldman E (1998). Recruitment variation during a pilot colonization study of hydrothermal vents.. Deep-Sea Res II.

[10] Kelly NE, Metaxas A, Butterfield DA (2007). Spatial and temporal patterns in colonization of deep-sea hydrothermal vent invertebrates on the Juan de Fuca Ridge, NE Pacific.. Aquat Biol.

[11] Shank TM, Fornari DJ, Von Damm KL, Lilley MD, Haymon RM (1998). Temporal and spatial patterns of biological community development at nascent deep-sea hydrothermal vents.. Deep-Sea Res II.

[12] Marcus J, Tunnicliffe V, Butterfield DA (2009). Post-eruption succession of macrofaunal communities at diffuse flow hydrothermal vents on Axial Volcano, Juan de Fuca Ridge, Northeast Pacific.. Deep-Sea Res II.

[13] Govenar B, Bergquist DC, Urcuyo IA, Eckner JT, Fisher CR (2002). Three *Ridgeia piscesae* assemblages from a single Juan de Fuca Ridge sulfide edifice: Structurally different and functionally similar.. Cah Biol Mar.

[14] Van Dover C L, Berg CJ, Turner RD (1988). Recruitment of marine invertebrates to hard substrates at deep-sea hydrothermal vents on the East Pacific Rise and Galapagos spreading center.. Deep-Sea Res A.

[15] Bernardino AF, Smith CR, Baco A, Altamira I, Sumida PYG (2010). Macrofaunal succession in sediments around kelp and wood falls in the deep NE Pacific and community overlap with other reducing habitats.. Deep-Sea Res I.

[16] Clarke KR, Warwick RM (1999). The taxonomic distinctness measure of biodiversity: weighting of step lengths between hierarchical levels.. Mar Ecol Progr Ser.

[17] Clarke KR, Warwick RM (2001). A further biodiversity index applicable to species lists: variation in taxonomic distinctness.. Mar Ecol Progr Ser.

[18] Metaxas A (2004). Spatial and temporal patterns in larval supply at hydrothermal vents in the northwest Pacific Ocean.. Limnol Oceanogr.

[19] Govenar B, Fisher CR (2007). Experimental evidence of habitat provision by the siboglinid polychaete *Riftia pachyptila*.. Mar Ecol.

[20] Brooke SD, Young CM (2009). Where do embryos of *Riftia pachyptila* develop? Pressure tolerances, temperature tolerances, and buoyancy during prolonged embryonic dispersal.. Deep Sea Res II.

[21] Marsh AG, Mullineaux LS, Young CM, Manahan DT (2001). Larval dispersal potential of the tubeworm *Riftia pachyptila* at deep-sea hydrothermal vents.. Nature.

[22] Pradillon F, Shillito B, Young CM, Gaill F (2001). Developmental arrest in vent worm embryos.. Nature.

[23] Herring PJ, Dixon DR (1998). Extensive deep-sea dispersal of postlarval shrimp from a hydrothermal vent.. Deep-Sea Res I.

[24] Van Gaest A (2006). Ecology and early life history of *Bathynerita naticoidea*: evidence for long-distance larval dispersal of a cold seep gastropod..

[25] Arellano SM (2008). Embryology, larval ecology, and recruitment of *"Bathymodiolus" childressi*, a cold seep mussel from the Gulf of Mexico..

[26] Arellano SM, Young CM (2009). Spawning, development, and the duration of larval life in a deep-sea cold-seep mussel.. Biol Bull.

[27] Rouse GW, Wilson NG, Goffredi SK, Johnson SB, Smart T, Widmer C (2009). Spawning and development in *Osedax* boneworms (Siboglinidae, Annelida).. Mar Biol.

[28] Beaulieu SE, Mullineaux LS, Adams DK, Mills SW (2009). Comparison of a sediment trap and plankton pump for time-series sampling of larvae near deep-sea hydrothermal vents.. Limnol Oceanogr Methods.

[29] Kelly N, Metaxas A (2006). Recruitment patterns of invertebrates at anhydrite hydrothermal vents on the Juan de Fuca Ridge, NE Pacific.. Cah Biol Mar.

[30] Mullineaux LS, Peterson CH, Micheli F, Mills SW (2003). Successional mechanism varies along a gradient in hydrothermal fluid flux at deep-sea vents.. Ecol Monogr.

[31] Micheli F, Peterson CH, Mullineaux LS, Fisher CR, Mills SW (2002). Predation structures communities at deep-sea hydrothermal vents.. Ecol Monogr.

[32] Hunt HL, Metaxas A, Jennings RM, Halanych KM, Mullineaux LS (2004). Testing biological control of colonization by vestimentiferan tubeworms at deep-sea hydrothermal vents (East Pacific Rise, 9°50’N).. Deep-Sea Res I.

[33] Levin LA, Ziebis W, Mendoza GF, Growney-Cannon V, Walther S (2006). Recruitment response of methane-seep macrofauna to sulfide-rich sediments: an in situ experiment.. J Exp Mar Biol Ecol.

[34] Grassle JF (1977). Slow recolonization of deep-sea sediment.. Nature.

[35] Grassle JF, Morse-Porteous LS (1987). Macrofaunal colonization of disturbed deep-sea environments and the structure of deep-sea benthic communities.. Deep-Sea Res A.

[36] Smith CR, Hessler RR (1987). Colonization and succession in deep-sea ecosystems.. TREE.

[37] Baco AR, Smith CR (2003). High species richness in deep-sea chemoautotrophic whale skeleton communities.. Mar Ecol Prog Ser.

[38] Turnipseed M, Knick KE, Lipcius RN, Dreyer J, Van Dover CL (2003). Diversity in mussel beds at deep-sea hydrothermal vents and cold seeps.. Ecol Lett.

[39] Scott KM, Fisher CR (1995). Physiological ecology of sulfide metabolism in hydrothermal vent and cold seep vesicomyid clams and vestimentiferan tube worms.. Am Zool.

[40] Tsurumi M, Tunnicliffe V (2003). Tubeworm-associated communities at hydrothermal vents on the Juan de Fuca Ridge, northeast Pacific.. Deep-Sea Res I.

[41] Distel DL, Baco AR, Chuang E, Morrill W, Cavanaugh C, Smith CR (2000). Do mussels take wooden steps to deep-sea vents?. Nature.

[42] Khripounoff A, Vangriesheim A, Crassous P, Segonzac, Lafon V (2008). Temporal variation of currents, particulate flux and organism supply at deep-sea hydrothermal fields of the Azores Triple Junction.. Deep-Sea Res I.

[43] Haderlie EC (1983). Depth distribution and settlement times of the molluscan wood borers *Bankia setacea* (Tryon, 1863) and *Xylophaga washingtona* Bartsch, 1921, in Monterey Bay.. The Veliger.

[44] Berg CJ, Butman B, Early JA, Turner RD (1987). Seasonal recruitment of marine invertebrates to hard substrates on Georges Bank and the eastern continental shelf of the United States.. The Nautilus.

[45] Romey WL, Castro KM, Dealteris JT, Bullock RC (1991). Recruitment in the deep-sea wood-boring bivalve *Xylophaga atlantica* Richards.. The Veliger.

[46] Tyler PA, Young CM, Dove F (2007). Settlement, growth and reproduction in the deep-sea wood-boring bivalve mollusc *Xylophaga depalmai*.. Mar Ecol Progr Ser.

[47] Khripounoff A, Vangriesheim A, Crassous P, Segonzac M, Colaco A (2001). Particle flux in the Rainbow hydrothermal vent field (MId-Atlantic Ridge): dynamics, mineral and biological composition.. J Mar Res.

[48] Kelly NE, Metaxas A (2008). Diversity of invertebrate colonists on simple and complex substrates at hydrothermal vents on the Juan de Fuca Ridge.. Aquat Biol.

[49] Tunnicliffe V, Embley RW, Holden JF, Butterfiled DA, Massoth GJ (1997). Biological colonization of new hydrothermal vents following an eruption on Juan de Fuca Ridge.. Deep-Sea Res I.

[50] Nussbaumer AD, Fisher CR, Bright M (2006). Horizontal endosymbiont transmission in hydrothermal vent tubeworms.. Nature.

[51] Pradillon F, Zbinden M, Le Bris N, Hourdez S, Barnay A-S (2009). Development of assemblages associated with alvinellid colonies in the walls of high-temperature vents at the East Pacific Rise.. Deep-Sea Res II.

[52] Watanabe H, Fujikura K, Kinoshita G, Yamamoto H, Okutani T (2009). Egg capsule of *Phymorhynchus buccinoides* (Gastropoda: Turridae) in a deep-sea methane seep site in Sagami Bay, Japan.. VENUS.

[53] Turner RD (1973). Wood-boring bivalves, opportunistic species in the deep sea.. Science.

[54] Voight JR (2007). Experimental deep-sea deployments reveal diverse Northeast Pacific wood-boring bivalves of Xylophagainae (Myoida: Pholadidae).. J Moll Stud.

[55] Lorion J, Duperron S, Gros O, Cruaud C, Samadi S (2009). Several deep-sea mussels and associated sumbionts are able to live both on wood and on whale falls.. Proc Royal Soc B Biol Sci.

[56] Fujiwara Y, Kawato M, Yamamoto T, Yamanaka T, Sato-Okoshi W (2007). Three-year investigations into sperm whale-fall ecosystems in Japan.. Mar Ecol.

[57] Braby CE, Rouse GW, Johnson SB, Jones WJ, Vrijenhoek RC (2007). Bathymetric and temporal variation among *Osedax boneworms* and associated megafauna on whale-falls in Monterey Bay, California.. Deep Sea Res I.

