# Supplementary material for: Do Larval Supply and Recruitment Vary among Chemosynthetic Environments of the Deep Sea?
Source: PLoS One. 2010 Jul 19;5(7):e11646. doi: 10.1371/journal.pone.0011646 (PMC2906503; doi:10.1371/journal.pone.0011646)
Supplement: Table S1 — Studies. JdF: Juan de Fuca Ridge; Exp: Explorer Ridge; EPR: East Pacific Rise; GAL: Galapagos Rift; MAR: Mid Atlantic Ridge; Gulf of Mexico; SCB: Santa Cruz Basin; CM: Cape Nomamisaki; MC: Monterey Canyon; NWA: Northwest Atlantic; NEP: Northeast Pacific; mab: metres above bottom; † these additional studies were used for taxonomic comparisons only. (0.11 MB DOC) [file pone.0011646.s001.doc]

Table S1: Studies. JdF: Juan de Fuca Ridge; Exp: Explorer Ridge; EPR: East Pacific Rise; GAL: Galapagos Rift; MAR: Mid Atlantic Ridge; Gulf of Mexico; SCB: Santa Cruz Basin; CM: Cape Nomamisaki; MC: Monterey Canyon; NWA: Northwest Atlantic; NEP: Northeast Pacific; mab: metres above bottom; ^†^ these additional studies were used for taxonomic comparisons only

| Life history process | Habitat | Location | Depth (m) | Sample type | Deployment (d) | Reference |
| --- | --- | --- | --- | --- | --- | --- |
|  |  |  |  |  |  |  |
| Larval supply | Vent | JdF; 45º56′N 130º00′W | 1550 | Larval tubes; 0.5 mab | 6-13 | [18] |
|  | Vent | EPR; 9º50′N 104º17.5′W | 2500 | Sediment traps; 4 mab | 10 | [8] |
|  | Vent | MAR; 36º14′N 33º54′W | 2250 | Sediment traps; 1.5 mab | 4 | [47] |
|  | Vent | MAR; 36º14′N 33º54′W | 2250 | Sediment traps; 2.5 mab | 10-15 | [42] |
|  | Vent | MAR; 37º17′N 32º17′W | 1600 | Sediment traps; 2.5 mab | 10-15 | [42] |
|  | Seeps | GoM; 27º43′N 91º16′W | 650 | Larval tubes; 0.3 mab | 240-270 | [25] |
|  |  |  |  |  |  |  |
| Settlement | Vent | JdF; 45º56′N 129º05′W | 1550 | Colonization panels (basalt) | NA | [10] |
|  | Vent | JdF; 47º57′N 129º05′W | 2200 | Colonization panels (basalt) | NA | [10] |
|  | Vent | EPR; 9º50′N 104º17.5′W | 2500 | Colonization blocks (basalt) | NA | [9] |
|  |  |  |  |  |  |  |
| Recruitment | Vent | JdF; 45º56′N 129º05′W | 1550 | Colonization panels (basalt) | 352-410 | [10] |
|  | Vent | JdF; 47º57′N 129º05′W | 2200 | Colonization panels (basalt) | 350-357 | [10] |
|  | Vent | JdF; 45º56′N 129º05′W | 1550 | Colonization panels (sponge) | 352-410 | [48]^†^ |
|  | Vent | JdF; 45º56′N 129º05′W | 1550 | Suction samples  (after eruption) | 210-540 | [12] |
|  | Vent | JdF; 47º57′N 130º00′W | 2200 | Rocks (in diffuse flow) | 300 | Levin LA (unpub) |
|  | Vent | JdF; 46º17′N 129º40′W | 2280 | Tubeworm bushes and suction samples  (after eruption) | 365 | [49]^†^ |
|  | Vent | GAL; 0º48′N 86º09′W | 2450 | Colonization panels (slate) | 260-321 | [14] |
|  | Vent | EPR; 20º50′N 109º06′W | 2600 | Colonization panels (slate) | 23-26 | [14] |
|  | Vent | EPR; 9º50′N 104º17.5′W | 2500 | Photographic and video surveys (after eruption) | 326 | [11] |
|  | Vent | EPR; 9º50′N 104º47′W | 2500 | Colonization blocks (basalt); vestimentiferan zone | 240 | [31] |
|  | Vent | EPR; 9º50′N 104º47′W | 2500 | Colonization blocks (basalt); vestimentiferan and bivalve zones | 390 | [30] |
|  | Vent | EPR; 9º50′N 104º17.5′W | 2500 | Tubeworm bushes after clearance experiment | 326 | [13] |
|  | Vent | EPR; 9º50′N 104º17.5′W | 2500 | Artificial tubeworm bushes | 316 | [19] |
|  | Vent | EPR; 9º50′N 104º17.5′W | 2500 | Panels (basalt) | 293 | Shank T (unpubl) |
|  | Vent | EPR; 9º50′N 104º17.5′W | 2500 | Artificial colonization cubes (HPVC) | 365 | [50]^†^ |
|  | Vent | EPR; 9º50′N 104º17.5′W  12º48.5′N 103º56′W | 2500  2600 | Artificial colonization tubes | 5-224 | [51]^†^ |
|  | Seeps | Eel River; 47º6′N 135º42′W | 525 | Colonization trays (with added sulphide addition, in seeps) | 180 | [33] |
|  | Seeps | Japan; 35º00′N 139º14′E | 1170 | Molluscan shells | NA | [52]^†^ |
|  | Wood | Monterey Bay; 36º41′N-36º45′N 121º58′W-122º02′W | 100-500 | Panels | 120-180 | [43] |
|  | Wood | Georges Bank; 40º29′N 70º30′W, 40º02′N 67º34′W, | 64-100 | Panels | 120-180 | [44] |
|  | Wood | Cape Cod; 40º26′N 70º28′W, 39º55′N 69º44′W | 100-200 | Panels | 29-212 | [45] |
|  | Wood | Bahamas; 25º53′N 77º32′W | 480-520 | Panels | 59-183 | [46] |
|  | Wood | SCB; 33º30′N 119º22′W | 1670 | Sediment cores (0 m from wood) | 180-660 | [15] |
|  | Wood | JdF; 47º57′N 130º00′W | 2200 | Wood blocks (no flow) | 300 | Levin LA (unpub) |
|  | Wood | NWA; 39º46′N 70º41′W | 1830 | Wood blocks (no flow) | 104 | [53]^†^ |
|  | Wood | NEP; 41-50ºN 126-130ºW | 1520-3232 | Wood blocks (no flow) | 365 | [54]^†^ |
|  | Wood | Vanuatu; 15º42′S 167º02′E | 441 | Wood | 365 | [55]^†^ |
|  | Kelp | SCB; 33º30′N 119º22′W | 1670 | Sediment cores (0 m from kelp) | 180-660 | [15] |
|  | Whale | CM, Japan; 31º20′N 129º59′W | 219-254 | Bone | 534 | [56] |
|  | Whale | SCB; 33º30′N 119º22′W | 1670 | Sediment cores (0 m from whale) | 660 | Smith CR (unpub) |
|  | Whale | JdF; 47º57′N 130º00′W | 2200 | Whale bones (no flow) | 300 | Levin LA (unpub) |
|  | Whale | MC; 36º42′N 122º05′W | 385, 1018 | Implanted whale carcass | 121-395 | [57]^†^ |
|  | Whale | Vanuatu; 15º42′S 167º02′E | 441 | Whale bones | 365 | [55]^†^ |
